# Protocol: a method to study the direct reprogramming of lateral root primordia to fertile shoots

**DOI:** 10.1186/s13007-016-0127-5

**Published:** 2016-05-12

**Authors:** Abdul Kareem, Dhanya Radhakrishnan, Xin Wang, Subhikshaa Bagavathiappan, Zankhana B. Trivedi, Kaoru Sugimoto, Jian Xu, Ari Pekka Mähönen, Kalika Prasad

**Affiliations:** School of Biology, Indian Institute of Science Education and Research, Thiruvananthapuram, Kerala 695016 India; Institute of Biotechnology, University of Helsinki, 00014 Helsinki, Finland; Department of Biosciences, Viikki Plant Science Centre, University of Helsinki, 00014 Helsinki, Finland; Department of Applied Biological Science, Faculty of Science and Technology, Tokyo University of Science, 2641 Yamazaki, Noda, Chiba 278-8510 Japan; Department of Biological Sciences and Centre for BioImaging Sciences, National University of Singapore, Singapore, 117543 Singapore

**Keywords:** *Arabidopsis*, Auxin, Cytokinin, Lateral root, Shoot, Regeneration, *Trans*-differentiation

## Abstract

**Background:**

Plants have the remarkable property to elaborate entire body plan from any tissue part. The conversion of lateral root primordium (LRP) to shoot is an ideal method for plant propagation and for plant researchers to understand the mechanism underlying *trans*-differentiation. Until now, however, a robust method that allows the efficient conversion of LRP to shoot is lacking. This has limited our ability to study the dynamic phases of reprogramming at cellular and molecular levels.

**Results:**

Here we present an efficient protocol for the direct conversion of LRP to a complete fertile shoot system. This protocol can be readily applied to the various ecotypes of *Arabidopsis*. We show that, the conversion process is highly responsive to developmental stages of LRP and changes in external environmental stimuli such as temperature. The entire conversion process can be adequately analyzed by histological and imaging techniques. As a demonstration, using a battery of cell fate specific markers, we show that confocal time-lapse imaging can be employed to uncover the early molecular events, intermediate developmental phases and relative abundance of stem cell regulators during the conversion of LRP to shoot.

**Conclusion:**

Our method is highly efficient, independent of genotypes tested and suitable to study the reprogramming of LRP to shoot in intact plants as well as in excised roots.

**Electronic supplementary material:**

The online version of this article (doi:10.1186/s13007-016-0127-5) contains supplementary material, which is available to authorized users.

## Background

The profound capacity of somatic cells to reproduce an entire organism has been widely exploited in in vitro plant regeneration [1, 2]. This spectacular property of plant cells has great impact on both, basic plant research and biotechnological application. On the one hand, in vitro plant regeneration can be exploited to understand many fundamental questions pertaining to subjects such as stem cell specification, cell fate determination, cell differentiation, meristem formation and organogenesis in the absence of positional information laid down during embryogenesis. On the other hand, the relatively simple and robust approach of this system attracts biotechnology sector for in vitro micropropagation, haploid production and genetic engineering.

Regeneration can be induced from the explant either directly or indirectly and are defined based on the presence or absence of an intermediate phase that leads to the formation of callus, a regenerative mass of cells. Indirect regeneration demands pre-treatment of the explant with high concentration of auxin which induces callus formation. Callus formation involves the activation of pericycle or pericycle-like cells and largely follows lateral root developmental pathway regardless of the origin of explants [3–5]. Root or shoot can be regenerated from callus when it is cultivated on media containing high auxin relative to cytokinin or high cytokinin relative to auxin, respectively. On the other hand, organs can also be regenerated directly from the explants without passing through a callus phase. Direct conversion of lateral root primordia (LRP) to shoot upon cytokinin treatment is one such example [4, 6, 7]. LRP is formed from xylem pole pericycle cells and it is induced by auxin [8, 9]. Application of cytokinin can convert LRP to shoot in a concentration-dependent manner and this process provides an elegant example of *trans*-differentiation [4, 6, 7]. Cytokinin rapidly induces the expression of a shoot stem cell regulator, *WUSCHEL* (*WUS*) in the LRP, and facilitates the conversion process [6, 7]. Consistent with the rapid upregulation of *WUS* during cytokinin-induced LRP-to-shoot conversion, inducible ectopic overexpression of *WUS* can convert LRP to shoot in the absence of external inductive cues and can induce shoot formation from *Arabidopsis* primary root meristem [6, 10].

Direct conversion of LRP to shoot can be exploited in sterile plants and in those where normal propagation by seed is not possible. This approach can substitute callus-mediated shoot regeneration that is more time consuming, laborious and difficult to achieve in some plant species. Moreover, explants that undergo prolonged sub-culture might encounter somaclonal variation associated with callus growth, and thus, such long-term callus-mediated culturing may not be desirable for the regeneration of elite crop species [11]. Therefore, direct conversion of LRP to shoot offers an attractive alternative for plant breeding industry.

**Fig. 1:**
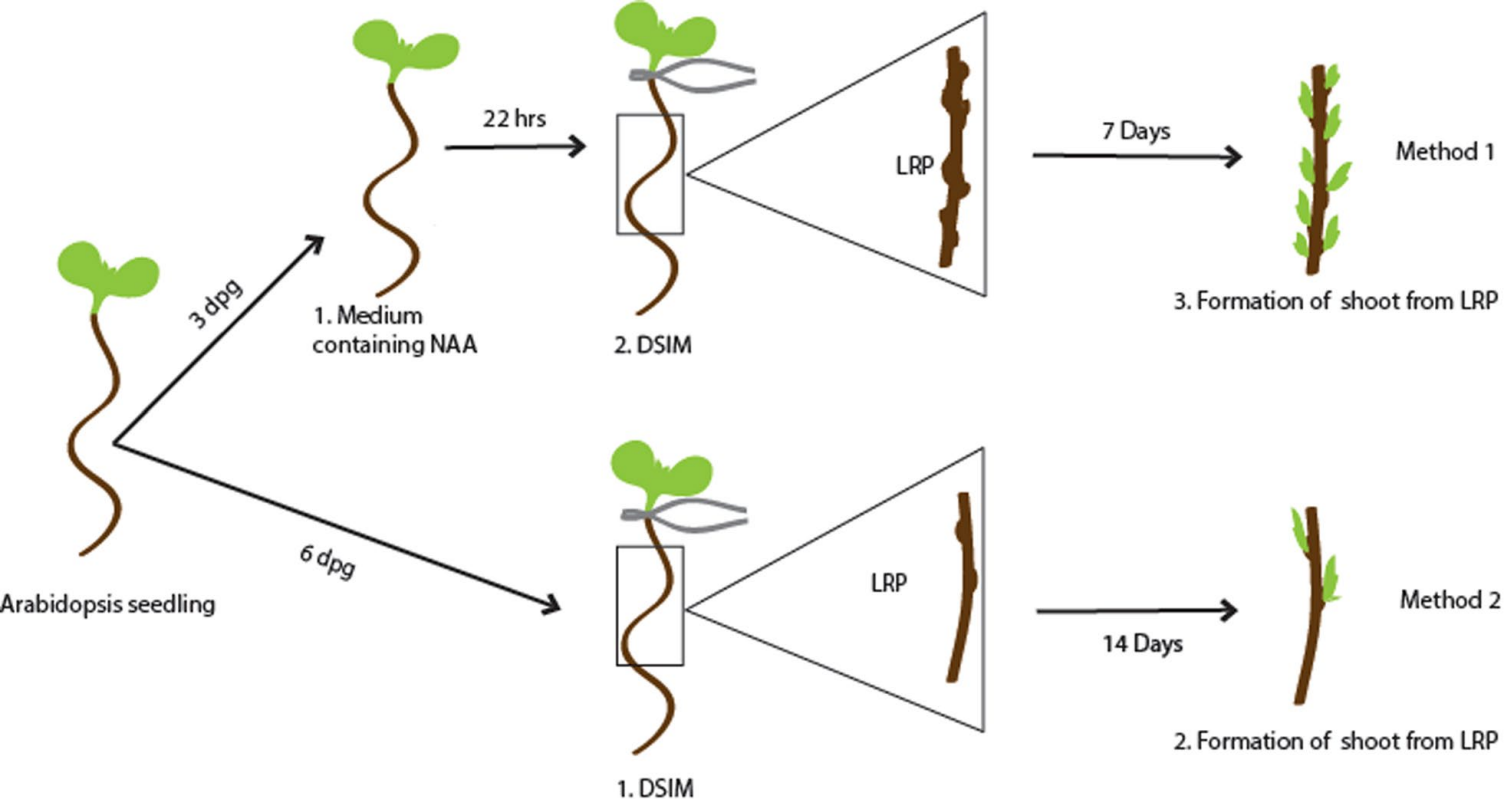
Schematic representation of direct conversion of LRP to shoot in *Arabidopsis thaliana*

Conversion of LRP to shoot must involve massive reprogramming in response to shoot inductive cues. Study of early cellular and molecular events regulating the reprogramming is essential to probe the underlying mechanisms. The model dicot species, *Arabidopsis*, can be used to study the in depth mechanism and it can serve as a reference for studies in other plant species [12–14]. Recent studies in *Arabidopsis* have begun to address this process [4, 6, 7]. Although previous studies in *Arabidopsis* have attempted to understand the molecular players involved in LRP-to-shoot conversion, they do not provide a robust method that can monitor the transient intermediate phases of conversion in real time [4, 7]. Dissection of the intermediate phases in which the converting structures are likely to remain morphologically identical to untreated LRP is instrumental to understand the mechanisms. Here, we describe a simple and highly reproducible method that can be used for the synchronized and efficient conversion of LRP to complete fertile shoot system in *Arabidopsis* which allows us to dissect the transient intermediate developmental phases of conversion (Fig. 1). We reveal the unrecognized role of external environmental stimulus, temperature, in the conversion of LRP to shoot. Using a battery of cell fate specific fluorescent markers, we show that time-lapse imaging is a useful tool to gain deeper insights into these processes. Furthermore, we illustrate the use of classical histological analysis to understand the anatomical changes during the conversion. Our methods can be used to study the process in both, the excised root as well as in growing plants.

## Materials

### Reagents

- Reagents for seed sterilization
  Dried seeds of *Arabidopsis thaliana*, 20 % sodium hypochlorite, 70 % ethanol and autoclaved distilled water.
- Murashige and skoog (MS) medium
  Use de-ionized Milli Q water to prepare growth media. All the hormones and antibiotics should be filter-sterilized and should be added to autoclaved, cool medium (45–50 °C). Preparation of hormone stocks is described in Table 1.

**Table 1:** Details of hormone/antibiotic stock preparation

Antibiotic/hormone	Stock concentration	Solvent	Concentration in media	Amount to be added for 50 ml medium (µl)	Amount to be added for 1 l medium (ml)
Ampicillin	100 mg/ml	40 % ethanol	100 µg/ml	50	1
NAA	1.86 mg/ml	DMSO	10 µM	50	1
2iP	10 mM	DMSO	10 µM	50	1
Trans zeatin	10 mM	DMSO	10 µM	50	1
  To prepare 1L MS medium, add 2.165 g (for half strength) or 4.3 g (for full strength) of MS salt (Sigma, M5524) and 10 g of sucrose (Sigma, S0389). Adjust pH to 5.8 with 1 N KOH and add 8 g of plant Agar (Sigma, A7921). Autoclave the medium (121 °C for 20 min) and cool it to 45–50 °C. Add 1 ml of 100 mg/ml filter-sterilized Ampicillin (final conc. 100 µg/ml) to 1L medium and pour 50 ml each into square Petri dishes within the LAF.
  *Note* Addition of Ampicillin to the medium can prevent bacterial contamination.
- NAA pretreatment medium
  Add 50 µl of 1.86 mg/ml NAA (naphthaleneacetic acid) (final concentration 10 µM) to 50 ml full strength MS agar medium and pour into square Petri dishes in the LAF.
- Direct shoot inducing medium (DSIM)
  Add 4.3 g of MS salt (Sigma, M5524) and 10 g of Sucrose (Sigma, S0389), 0.5 g of MES (2-(*N*-Morpholino) ethane sulfonic acid) (Sigma, M2933) and 1 ml of Gamborg’s vitamin solution (Sigma, G1019) for 1 l of 1× DSIM. Adjust pH to 5.7 with 1 N KOH and add 8 g of plant Agar (Sigma, A7921). After autoclaving, cool the medium to 45–50 °C and add 1 ml of 100 mg/ml filter-sterilized Ampicillin (final conc. 100 µg/ml) and specific hormones to 1 l medium as mentioned below. Pour 50 ml medium into each square Petri dish in the LAF. Two hormonal combinations for DSIM are described here.
  DSIM-1: 10 µM 2iP (6-(γ,γ-dimethylallylamino) purine)
  DSIM-2: 10 µM *trans*-zeatin
- Root inducing medium (RIM)
  Add 500 µl of 1 mg/ml indol-3-butyric acid (IBA) into the basal composition of DSIM (without cytokinin) to make 1 l RIM. Pour into square Petri dishes in the LAF.
- Soilrite–vermiculite mixture
  Mix soilrite and exfoliated vermiculite (1:1) with 1× MS solution (without sucrose and agar) for *ex vitro* establishment of seedlings. It is desirable to autoclave both soilrite and vermiculite before mixing them with MS solution to destroy germs and weeds.
- Hormone/antibiotic stocks
  The hormone stocks should be prepared in DMSO and filter sterilized by 0.22 µm filter. All the stocks should be stored at −20 °C.
- Staining reagents for confocal microscopy
  10 µg/ml propidium iodide (filter sterilized) (Sigma).
  10 µg/ml FM4-64 (filter sterilized) (molecular probes).
  Both propidium iodide and FM4-64 should be dissolved in sterile distilled water followed by filter sterilization. Store in amber coloured tubes at 4 °C.
  *Note* Caution! Propidium iodide is a potential carcinogen. Wear protective gloves to avoid contact with skin.
- Fixing solution preparation for histological analysis
  401 ml ddH_2_O
  25 ml 1 M Na_3_PO_4_ with pH 7.2
  54 ml 37 % formaldehyde
  20 ml 25 % glutaraldehyde
  *Note* Formaldehyde and glutaraldehyde are toxic. The preparation should be performed in fume hood.
- Solution A preparation
  Leica historesin embedding kit is used for embedding. Dissolve 0.5 g historesin activator into 50 ml basic resin, mix thoroughly. Solution A can be stored at 4 °C, dark, for up to 2 weeks.
- Embedding solution preparation
  Add hardener into solution A with the volume ratio 1:14. Mix thoroughly by vortexing.
  *Note* Embedding solution should be made freshly before use as it cannot be stored. It hardens within 10 min.
- Plant materials and constructs
  *Arabidopsis* ecotypes Col-0, Ws and L*er* are used in this study. *pPLT2::PLT2*-*vYFP* [6, 15] *pSCR::H2B*-*vYFP*, and *pWUS::erCFP* [6] have been described previously. *pARF10::ARF10*-*YFP* construct is generated by incorporating genomic sequence of *ARF10* between its 4.984 kb upstream regulatory sequence at the 5′ end and the coding region of *vYFP* at the 3′ end. The genomic sequence of *STM* is cloned with its 5.752 kb upstream regulatory sequence fused in translational frame with the *vYFP* to generate *pSTM::STM*-*YFP*. All the DNA fragments mentioned above are amplified from Col-0 genomic DNA using the primers listed in Table 2. Multisite Gateway recombination (Invitrogen) based cloning system is used to clone all components into pCAMBIA 1300 destination vector. Floral-dip method is used to transform Col-0 plants and transgenics are selected based on hygromycin resistance.

**Table 2:** Details of oligonucleotide primers (gateway compatible) used in this study (5′–>3′)

Primer name	Forward primer	Reverse primer
*ARF10* promoter	ggggacaactttgtatagaaaagttgttgtcacgattagaatgcacatgcagttcgt	ggggactgcttttttgtacaaacttgtctagacgaagttgtgtaacccccaaattct
*ARF10* gene	ggggacaagtttgtacaaaaaagcaggctgtatggagcaagagaaaagcttggatccacaac	Ggggaccactttgtacaagaaagctgggttagcgaagatgctgagcggaccagtcttcgtag
*STM* gene	ggggacaagtttgtacaaaaaagcaggctgtatggagagtggttccaacagcacttcttgtc	Ggggaccactttgtacaagaaagctgggttaagcatggtggaggagatgtgatccattgg

### Equipment

#### Equipment for in vitro culture

- Laminar air flow chamber (LAF)
- Microscissors-Vannas scissor, straight (Ted Pella, 1346) (flame sterilized)
- Forceps (flame sterilized)
- Scalpel and scalpel blades (flame sterilized)
- Micro pipettes
- Sterile pipette-tips (200 µl and 1 ml)
- 1.5 ml micro-centrifuge tubes
- Spirit lamp
- Sterile disposable square Petri plates, size: 120 mm × 120 mm (Himedia PW050-1)
- Parafilm
- Gloves
- Plant growth chamber (Percival AR-100L3)

#### Equipment for microscopy

- Stereo microscope (Leica M205FA) for bright field imaging
- Confocal laser scanning microscope (Leica TCS SP5 II) for time-lapse fluorescent imaging
- Microscope slides (Himedia CG029)
- Microscope cover glass 22 × 22 mm (Corning 2850-22)

#### Equipment for resin cross section

- Micro pipettes
- Sterile pipette tips (200 µl and 1 ml)
- 1.5 ml micro centrifuge tubes
- 13 × 19 × 5 mm Polyethylene mold cups
- Flat bottom 6 well cell culture plates
- 20 × 15 × 10 mm wooden block
- Microscope slides
- Microtome (Leica, Jung RM2055)
- Microtome knife
- Water bath (Kunz instruments, HIS-2)
- Flattening table (Kunz instruments, HP-3)
- Copier films (Folex, A4 size)
- Double side adhesive tape
- Scissors and forceps

### Equipment setup

- Embedding chamber making
  Cut the copier film into size of 3 × 4 cm, cover it with double side adhesive tape, then dig a hole in the middle with the size 1 × 2 cm, stick it to another piece of intact copier film, the chamber left can be used to embed material. The chamber depth equals to the film thickness. If the material is thick, just stick two or more layers of film together to make the chamber deeper.
  *Note* This method is quite flexible, this can be modified by using another material instead of copier film, as long as the material is not organic solution permeable and if a chamber to hold the samples can be made. The chamber size can be adjusted according to the sample size. This kind of chamber is recyclable.

### Methods

#### Seed sterilization and inoculation

A sterile working environment should be ensured during all steps of plant tissue culture, from seed sterilization to shoot regeneration. Clean the working area of LAF and the surfaces of necessary materials/tools (petri plates, pipettes, tip box, forceps, scissors, spirit lamp and bottles containing ethanol, bleach and sterile water) with 70 % ethanol. Flame-sterilize forceps and scissors before contact with each explant. Sterilize the LAF and the materials/tools by UV irradiation for 20 min. Hands should be wiped with 70 % ethanol prior to the commencement of the work.

A liquid surface sterilization method for seeds is introduced here. The vapour phase method can also be used as described in Ref. [16].

1. Add 1 ml of 70 % ethanol to the seeds in 1.5 ml sterile micro-centrifuge tube.
  *Note* The maximum amount of seeds that can be effectively sterilized in a 1.5 ml microfuge tube is until 50 μl mark.
2. Gently mix it for 5 min by inverting the tubes followed by a brief spin at 6000 rpm.
3. Discard the ethanol and add 1 ml of 20 % bleach (sodium hypochlorite) and mix well for 5 min followed by a brief spin at 6000 rpm.
  *Note* It should be ensured that bleach treatment does not exceed maximum 10 min as it adversely affects seed germination.
4. Wash the seeds with 1 ml of sterile deionized water 5–7 times.
5. Store the sterilized seeds in a micro-centrifuge tube containing 1 ml water at 4 °C for 2–5 days for stratification.
  *Note* Stratification is important for synchronized seed germination. Stratification treatment can be provided even in plate after seed inoculation.
6. Pipette the sterilized seeds and inoculate over ½ MS agar plate in 2 rows. Spread the seeds distinctly in a linear manner using 1 ml sterile pipette tip. One row of square petri plate (120 mm × 120 mm) can accommodate approximately 30 seeds. Seal the plates with parafilm.
7. Incubate the plates in plant growth chamber maintained at 22 °C, 45 µmol/m^2^ s and 16 h light/8 h dark. The plates can be placed slanting in racks made into plastic trays.
8. Follow the seed germination and collect the explants for shoot regeneration after 3rd or 6th day post germination (dpg) depending upon which method is followed.
  *Note* Plants should not be subjected to any kind of stress like nutrient stress, water stress or contamination before and after the explant collection. This is because stress can affect the shoot conversion efficiency. LRP-to-shoot conversion is hyper sensitive to temperature. Any deviation in temperature affects root-to-shoot conversion. Therefore, it is necessary to ensure that the water, which accumulates at the bottom of the plates during incubation in growth chamber should be removed on periodic basis as it can lead to bacterial contamination.

#### Explant excision and LRP-to-shoot conversion

**Fig. 2:**
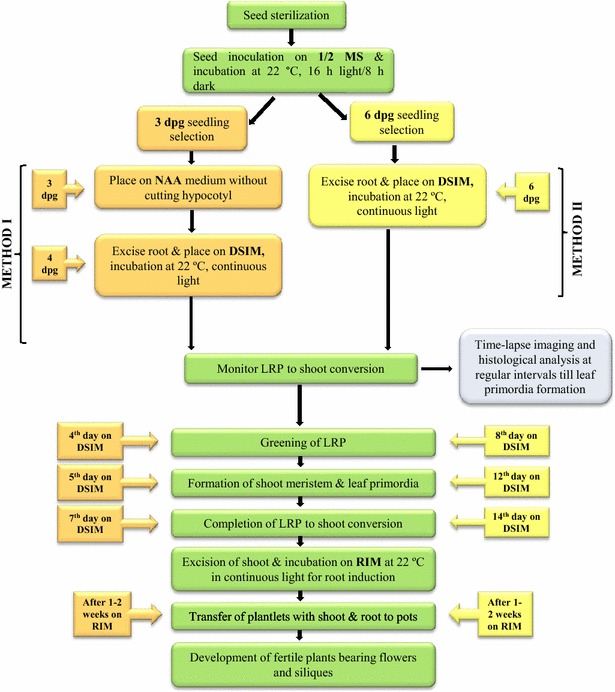
Schematic representation of the steps and time involved in direct conversion of LRP to shoot in *Arabidopsis thaliana*

In order to convert LRP directly into shoot, two methods based on Refs. [4, 6, 7] with modifications are introduced here. First method utilizes 3 dpg old seedlings and it requires a short auxin pre-treatment before transferring into cytokinin rich DSIM. The second method exploits 6 dpg old seedlings and the explants are directly transferred into DSIM (Fig. 2).

*Method-I* The two major advantages of the first method over the second method are its *speed* and *efficiency*. Method-1 is *speedy* as it utilizes very young (3 dpg) explants and the LRP-to-shoot conversion occurs within 6–7 days. This method is more *efficient* in converting maximum number of LRP into shoot. One of the disadvantages of this method is that it requires an additional step of NAA pre-treatment.

1. Transfer 3 dpg old seedlings from MS plate onto NAA (10 µM) containing medium. Seal the plate with parafilm and incubate for 20–22 h in plant growth chamber under the same conditions mentioned above. NAA pretreatment can ensure LRP formation throughout the root. LRP stages at II and III are more responsive towards cytokinin treatments. NAA treatment should not exceed 24 h as it promotes lateral root outgrowth which is less sensitive to cytokinin. LRP can be observed adjacent to one another in all directions throughout the root using DIC/confocal microscope. More number of LRP can be noticed at the basal meristem (transition zone) where auxin sensitivity is high.
  *Note* Pay attention to hold the seedlings at the cotyledon/leaf with flame-sterilized and cooled forceps during the transfer. Holding the seedlings at hypocotyl or root may damage the seedlings.
2. After 20–22 h of treatment on NAA-containing medium, transfer the seedlings onto DSIM using flame-sterilized and cooled forceps. Excise the root from the seedling by cutting at hypocotyl junction with flame-sterilized and cooled Vannas scissor. Root excision can be done on the medium. The wound scratches made on the medium while the excision of root will not affect conversion. Gently press the root explant on the medium using forceps so that the explant is in direct contact with the medium. Discard the shoot part along with hypocotyl.
  *Note* Use separate scissors and forceps for each seedling to avoid cross contamination. It is critical to ensure that the explants do not dry during these processes. The plate should not be kept open for a long time in LAF and the spirit lamp should be kept away from the plates to avoid the explants from drying.
3. Seal the plates with parafilm and incubate the same for 5–7 days under continuous light at 22 °C. The LRP will turn green in 4 days and shoot meristem and leaf primordia will be formed in 5 days. These can be viewed under high resolution microscope. LRP-to-shoot conversion will be completed by 7 days of incubation on DSIM. The developmental changes associated with LRP-to-shoot conversion can be monitored by time-lapse imaging under confocal microscope (see below). Newly formed leaves appear similar to the rosette leaves formed in seedlings with regular phyllotaxy and these exhibit trichomes on adaxial surface (Fig. 3). Narrow or filamentous leaves can also be observed occasionally.

**Fig. 3:**
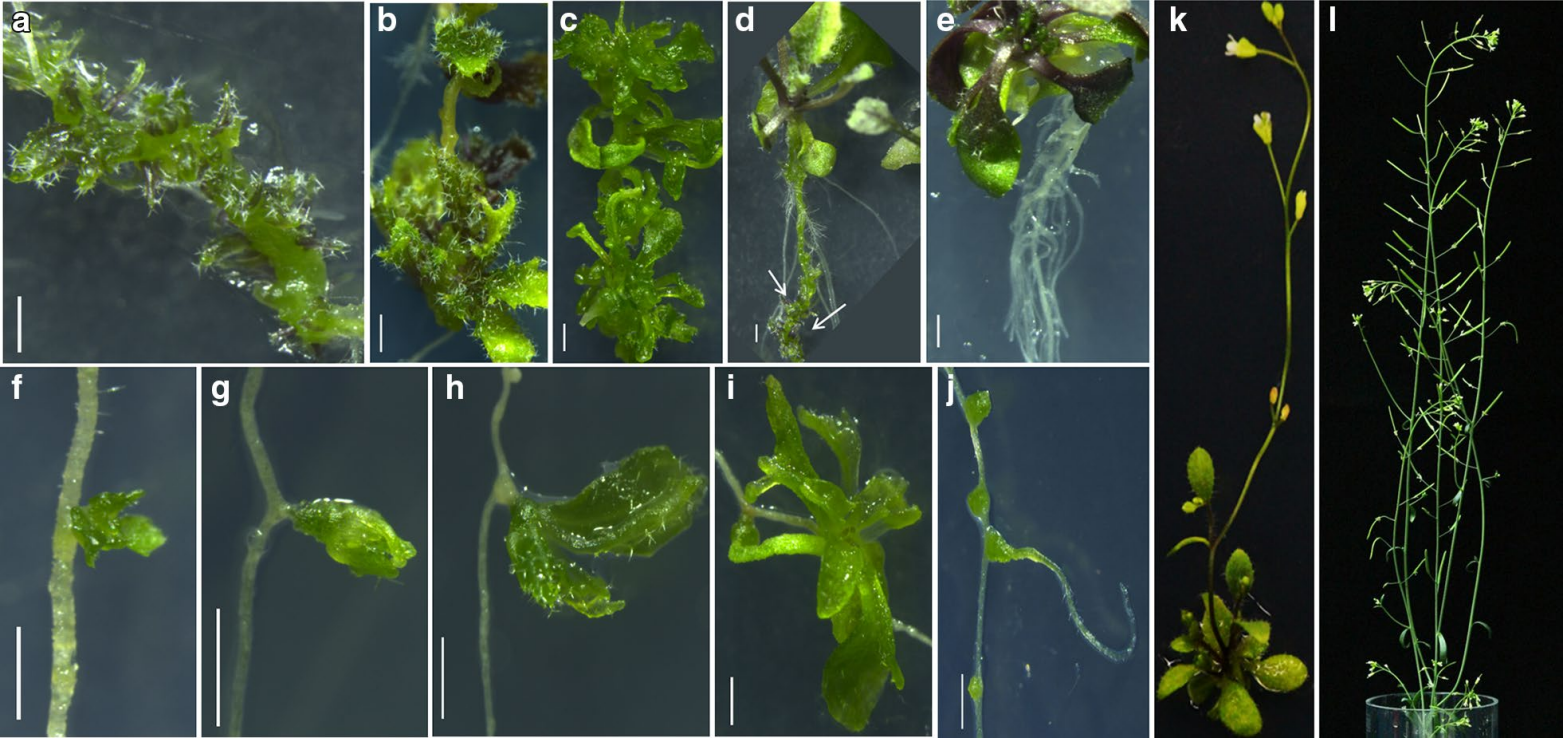
Conversion of LRP to complete fertile shoot system. **a**–**c** Stages of direct conversion of LRP to shoot by Method I after transfer from NAA to DSIM. **d** LRP-to-shoot conversion in growing plant after shoot inductive cues. *Arrows mark* converted shoots. **e** Root induction from regenerated shoot after incubation on RIM. **f**–**i** Direct LRP-to-shoot conversion by Method II after incubation on DSIM. **j** Root formation from the bulges developed in LRP at high temperature. **k** LRP converted shoot bearing rosette leaves and inflorescence. **l** *Ex vitro* established complete fertile shoot system bearing flowers and siliques. *Scale bar* 1 mm
4. Excise the converted shoot from the explant with flame-sterilized and cooled scalpel and place it on ½ MS medium and incubate for 4–5 more days to induce more number of leaves on the shoot. Transfer the plantlets further to RIM plate to make roots. Seal the RIM plate with parafilm and transfer to the growth chamber under continuous light at 22 °C.
  *Note* If the shoots are left undetached from root explant for long time, inflorescence stem will be formed with cauline leaves and flowers. But we suggest the excision of the shoot from the explant at 4–6 leaves stage and transfer to RIM plate because the shoots will be clustered otherwise.
5. Rooting can be noticed after 1 week of treatment on RIM. Transfer the plantlets having both shoot and root to soilrite–vermiculite mixture for *ex vitro* establishment. (Soilrite–vermiculite mixture is made wet with 1× MS solution). Cover the pot with saran wrap, to maintain humidity, and shift the pot in a tray into the growth chamber.
6. Gradually expose the plantlets to low humidity by progressive removal of the saran wrap after a few days. Add water to the tray containing the pots at regular interval and add ½ MS solution at every fifth round. The regenerated plants will be fertile with flowers and siliques. No watering is required once the siliques have matured.

*Method II* This method employs direct transfer of 6 dpg old root explants into DSIM for the conversion of LRP into shoot.

1. Pick 6 dpg old seedlings from ½ MS plate by holding the leaves with flame sterilized and cooled forceps and transfer onto DSIM. Gently press the root on the medium so that the entire explant is in contact with DSIM.
2. Excise the root from the seedling by cutting at hypocotyl junction using flame sterilized and cooled Vannas scissor. Discard the shoot part along with the hypocotyl.
3. Seal the plate with parafilm and incubate the same for 2 weeks under continuous light at 22 °C. The LRP will turn green by 8 days and shoot meristem and leaf primordia will be formed by 12 days. The developmental changes during LRP-to-shoot conversion can be monitored by time-lapse imaging. LRP-to-shoot conversion will be completed by 14 days of incubation on DSIM. Shoots will be arranged acropetally on root in an alternating fashion similar to lateral root formation.
4. Excise the shoot from the explant with flame-sterilized and cooled scalpel and place on ½ MS medium and incubate for 7 more days to induce more number of leaves on the shoot. Transfer the plantlets further to RIM plate to make roots. Seal the RIM plate with parafilm and transfer to growth chamber under continuous light at 22 °C.
5. Follow steps 5 and 6 of Method I for rooting and *ex vitro* establishment.

#### Histological analysis

For histological sectioning seedlings are fixed according to Ref. [17] and embedded using Leica Historesin kit according to Ref. [18]. In this method, samples are embedded in ‘sandwich’ chambers, which is particularly useful when the samples are small. This is a fast and versatile method, and many samples can be analysed in one section.

1. Add proper contents of fixing solution to the plate’s well, gently remove and soak the explants into the fixing solution. Seal the plates using parafilm and leave it overnight.
2. Discard the fixing solution, replace by ethanol gradients, starting from 10 %, then proceed to 30, 50, 70, 95, 100 %, and again 100 %, 30 min for each incubation. The material can be stored in 70 % ethanol.
3. Add equal volume of pure ethanol to solution A to get the half solution A. Immerse the explants in half solution A for at least 3 h. Lastly change to solution A for another 3 h. For older material, a longer incubation is recommended.
4. Gently place the explants in embedding chamber, use forceps to align them properly, make sure all explants are positioned in the same direction, add some drops of embedding solution, then carefully cover the chamber with another piece of film to insulate it from air. Make sure no air bubbles are left in the chamber. The sample-containing embedding solution will completely harden within 3 h.
5. Remove the cover film from the chamber, and take the sample containing solidified embedding piece out. Trim the embedding piece properly. Cut it into smaller pieces every 3 mm, and put them on top of each other to make a stack. All these pieces should be placed in the same direction and order relative to original explants. Add some drops of embedding solution to make them stick to each other. Their relative position should not be changed. In this way, you will get the results of more than one sample and more than one position in one section.
6. 3 h later, when the stack hardens, transfer the stack into the polyethylene mold cup (place the root containing stacks vertically for cross section and horizontally for longitudinal section). Add about 2 ml embedding solution to soak the stack and then cover a lid to insulate the air. Keep it over night before microtome.
7. Take the plastic block out from the mold cup. Trim it and stick it to a wooden block using super glue. Set the microtome machine, set the water bath temperature at 50 °C and the flattening table at 60 °C. Then make normal cross sections. Transfer each section piece to water bath to make it expand, then fish it out using a slide and dry the slide on the flattening table.
8. Immerse the slides in toluidine blue for 3–6 s and gently wash it using tap water. Add some drops of water on the slide and mount the slide. Observe under the microscope.

#### Confocal time-lapse imaging

Developmental changes during the conversion of LRP into shoot can be monitored by time-lapse imaging/live imaging of fluorescent protein tagged cell fate specific markers using confocal microscope. We are introducing a method to follow the time lapse imaging of the explant from untreated 0-day sample till complete shoot formation without contaminating the same. Live imaging can also be done using different explants to follow the developmental changes. The confocal settings we describe are based on Leica TCS SP5 II laser scanning microscope. The excitation/emission wavelengths will be invariable in all microscopes, but slight modifications may be required for laser power and intensity in different microscopes even if it is the same model. Imaging of the explants should commence before transferring to DSIM (method I: LRP of 20 h NAA treated explant. Method II: LRP of 6 dpg explant). Make sure that all materials used for time-lapse imaging are sterile. Microscope slides, cover glasses and pipette tips used during time-lapse imaging should be autoclaved. To avoid sticking together of cover glasses due to water vapour and to avoid the formation of water film during autoclaving, cover them individually in tissue paper and then wrap in an autoclavable, sealed polythene cover. Propidium iodide and FM4-64 should be filter-sterilized. (Dye concentration is given under materials section above).

1. Pipette 60–80 µl propidium iodide/FM4-64 onto clean microscope slide. Use propidium iodide for LRP imaging at 0-day and early days on DSIM. FM4-64 stain should be used for shoot meristem and shoot primordia imaging. Propidium iodide stains the cell wall while FM4-64 stains cell membrane to mark the cell boundaries. (Caution!: propidium iodide is a potential carcinogen, wear protective gloves during handling).
2. Aseptically transfer the explants from the respective medium into propidium iodide/FM4-64 on the slide using sterilized forceps.
3. Immerse the explants in propidium iodide/FM4-64 and stain for 2 min. This step is crucial to stain all cell files adequately.
4. Place the cover glass carefully over the sample without introducing air bubbles. The entire explant can be covered with 22 × 22 mm cover glass if the explant is kept in S shape. Hypocotyl and shoot part of 0-day explant should be kept out of the cover glass so that the root part is in single plane. However, ensure that the shoot part does not dehydrate during imaging. Cover glass with 24 × 60 mm which cover entire slide can also be used, but 0-day explants may not remain stable in single plane, therefore, it may interfere with single section imaging.
5. Keep the slide under the confocal microscope and focus the LRP/shoot meristem. Use 10× air or 20× oil objective for imaging. The confocal settings described here is based on our recent paper [6]. The settings for commonly used fluorescent signals are listed in Table 3.

**Table 3:** Details of the settings for commonly used fluorescent signals

Signal	Excitation wavelength (nm)	Emission wavelength range (nm)
CFP	458	465–515
GFP	488	495–530
YFP/VENUS	514	520–545
Propidium iodide	514/561	585–630
FM4-64	514/561	620–750
Chlorophyll autofluorescence	488/514/561	620–750
  The laser power and intensity may vary based on the nature of the marker tagged with fluorescent protein and the extent of staining. Use 20–30 % power for argon laser. Laser intensity should be minimal in order to reduce bleaching of the fluorescent signal and to avoid over saturation. Adjust the intensity from 5 to 30 % and also adjust the same with gain. Capture single middle section images for LRP at 0 day and for early days on DSIM. For imaging the shoot meristem and the leaf primordia, capture the Z-stack of all sections.
6. Once imaging is completed, carefully remove the coverglass from the slide. To remove the coverglass without damaging the explants, hold the slide slanting and add surplus of sterile water from one side, so that the coverglass will be washed off.
7. Carefully transfer the explant into a fresh petri plate containing sterile water to remove the propidium iodide/FM4-64 content. Gently swirl the plate for some time to ensure the proper removal of the stain. Pay attention to avoid any external source of contamination during these processes.
8. Transfer the explants to its respective medium and incubate in plant growth chamber until next imaging. Follow the imaging process at regular interval till the leaf primordia forms.

## Results and discussion

Here we provide detailed methods which can be readily used to study the conversion of LRP-to-shoot in the model organism, *Arabidopsis.* Both methods described here were effective in converting LRP into complete shoot system (Fig. 3a–l). We examined if shoot conversion can be achieved only in excised root or if this method can also be implemented to growing plants where complete root system is in the context of growing shoot. We found that our method can also be used to trigger shoot formation from LRP in intact plant (Fig. 3d). The ability to convert the LRP to shoot by external inductive cues in both intact plant and excised root is consistent with our previous study where inducible ectopic overexpression of shoot stem cell regulator, *WUS*, triggered shoot from LRP of both intact plant as well as excised root [6].

Though previous studies [4, 6, 7] have shown that LRP can be converted to shoot, it was not known if the developing shoot can be used to generate complete fertile shoot system. We show that the converted shoots bearing flowers and siliques can be successfully established *ex vitro* (Fig. 3k, l). Method I, which demanded auxin pre-treatment, displayed more number of LRP-to-shoot conversion. This is due to the presence of more number of LRP in the explants of method I than those of method II. As previously shown, NAA pre-treatment induced more number of LRP along the entire length of the root at both xylem pole pericycle [7, 19]. Upon DSIM treatment, these LRP converted into shoot. But it was not known if LRP at all stages can convert into shoot. We have tested the capacity of different stages of LRP to convert into shoot. We found that LRP stages II and III were more responsive to convert into shoot (Fig. 5a, e, i, m). LRP at the very late stage of development (lateral root emergence stage) was recalcitrant to convert into shoot (Additional file 1). The degree of shoot conversion ability of LRP at different developmental stages might be due to the varying sensitivity of LRP to cytokinin. It has been reported that stage V/VI LRP is least sensitive to cytokinin [20]. Thus, the developmental stage of LRP plays a significant role in determining the conversion process.

Besides the developmental stage of LRP, the concentration of cytokinin is also a vital factor to accomplish LRP-to-shoot conversion. At lower concentration, cytokinin perturbs LRP organization and thereby suppresses lateral root outgrowth [20, 21]. At higher concentration, cytokinin triggers *trans*-differentiation leading to the conversion of LRP to shoot. We have tested various concentrations of three cytokinins such as 6-(γ,γ-dimethylallylamino) purine (2iP), *trans*-zeatin (TZ) and kinetin (KT) to identify the most effective concentration of cytokinin that can convert maximum number of LRP to shoot. We found that both 2iP and TZ (10 and 20 µM) were more efficient to convert LRP into shoot than KT (Method-I: 2iP = 8 ± 0.57, TZ = 7.8 ± 0.87, KT = 4.1 ± 0.85). While 10 µM 2iP or TZ was sufficient to convert LRP to shoot in method I, 20 µM of 2iP or TZ was required in method II.

Different ecotypes can respond differently to the conversion of LRP to shoot. We have tested our methods in three different ecotypes of *Arabidopsis*, namely, Columbia (Col-0), Wassilewskija (Ws) and Landsberg *erecta* (L*er*). Both methods described here for LRP-to-shoot conversion were effective in all the three ecotypes with variations in shoot formation efficiency (Table 4). Ecotype Col-0 displayed the conversion of maximum number of LRP to shoot followed by L*er* and Ws.

**Table 4:** Frequency of LRP-to-shoot conversion that occurred in different *Arabidopsis* ecotypes (based on method 1, DSIM-1)

Ecotypes	Frequency of shoot regeneration (per explant)
Col-0	8.62 ± 0.83
Ler	6.5 ± 1.17
Ws	5.5 ± 0.38

In addition to the developmental stages of root and hormonal concentrations, external environmental stimuli may play a crucial role in LRP-to-shoot conversion. The culture temperature can be a potential factor that influences the conversion. To test this, we cultured the explants at both normal temperature (22 °C) and high temperature (29 °C). Many of the LRP cultured at high temperature arrested (42 %) during the conversion process despite the abundance of cytokinin (Fig. 3j). These arrested LRP made green bulges. Interestingly, some of these bulges eventually lead to the formation of lateral roots (Fig. 3j). Consistent with our observation, a recent study shows that seedlings grown at high temperature make more lateral roots [22]. Incubation at lower temperature (18 °C/20 °C cycle) marginally reduced the efficiency of LRP-to-shoot conversion (Additional file 2). Our data suggests that LRP-to-shoot conversion is hypersensitive to temperature and the LRP choose default root developmental pathway at high temperature even in the presence of high cytokinin. We have also tested the effect of continuous light and diurnal (16 h light/8 h dark) conditions on LRP to shoot conversion. We did not notice any significant difference in conversion between these conditions (Additional file 3).

**Fig. 4:**
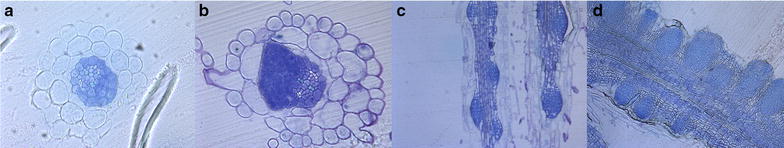
Anatomical changes during the conversion of LRP. **a**–**d** Histological section depicting the anatomical changes during the conversion of LRP to shoot meristem. **a** Cross section of a 3 dpg root treated with NAA for 20 h (i.e. first, lateral root induction step in method I). **b** Cross section of a 3 dpg root treated with NAA for 20 h followed by 4 day incubation on DSIM medium (i.e. second, shoot conversion step in method I). **c** Longitudinal section after lateral root induction step (3 days + 20 h NAA). **d** Longitudinal section after shoot conversion step (3 days + 20 h NAA + 4 days DSIM)

To study the anatomical changes during the LRP conversion to shoot meristem on DSIM, histological sections were generated (Fig. 4a–d). Our study shows that LRP-to-shoot conversion is accompanied by organized cell proliferation in LRP. These divisions, further, lead to the formation of shoot meristem and leaf primordium. Histological analysis can help to ascertain the cells in LRP that are competent to convert into shoot fate. Coupling these cellular changes with molecular changes can lead to the deeper insights into the *trans*-differentiation during LRP-to-shoot conversion.

**Fig. 5:**
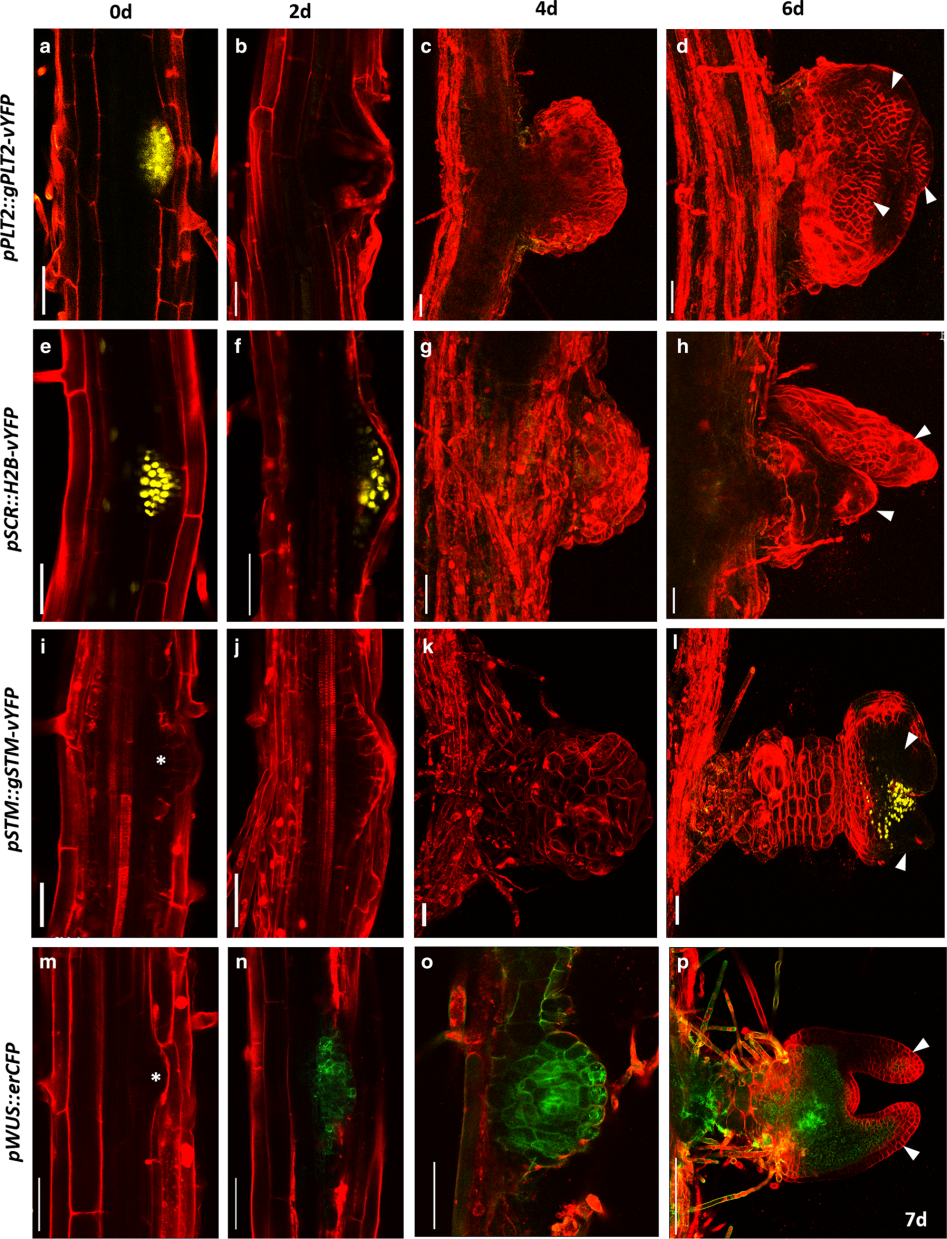
Confocal time lapse imaging during LRP to shoot conversion. **a**–**d** Time-lapse imaging showing *PLT2*-*vYFP* expression during the conversion of LRP to shoot. Note the down-regulation of *PLT2*-*vYFP* during the development of shoot meristem. **e**–**h** Dynamic expression pattern of *pSCR::H2B*-*vYFP* during the conversion of root to shoot. **i**–**l** Live imaging showing *STM*-*vYFP* expression during the shoot conversion. **l** Note the appearance of *STM*-*vYFP* in the nascent shoot meristem. **m**–**p** Spatiotemporal expression pattern of *pWUS::erCFP*. **p** Note the rapid upregulation of *pWUS::erCFP* upon cytokinin treatment. *Arrowhead* in (**d**, **h**, **l**, **p**) marks leaf primordium. *Red signal* is propidium iodide stain in (**a**, **b**, **e**, **f**, **i**, **j**, **m**, **n**) and FM4-64 stain in the remaining. *Scale bar* 50 µm

**Fig. 6:**
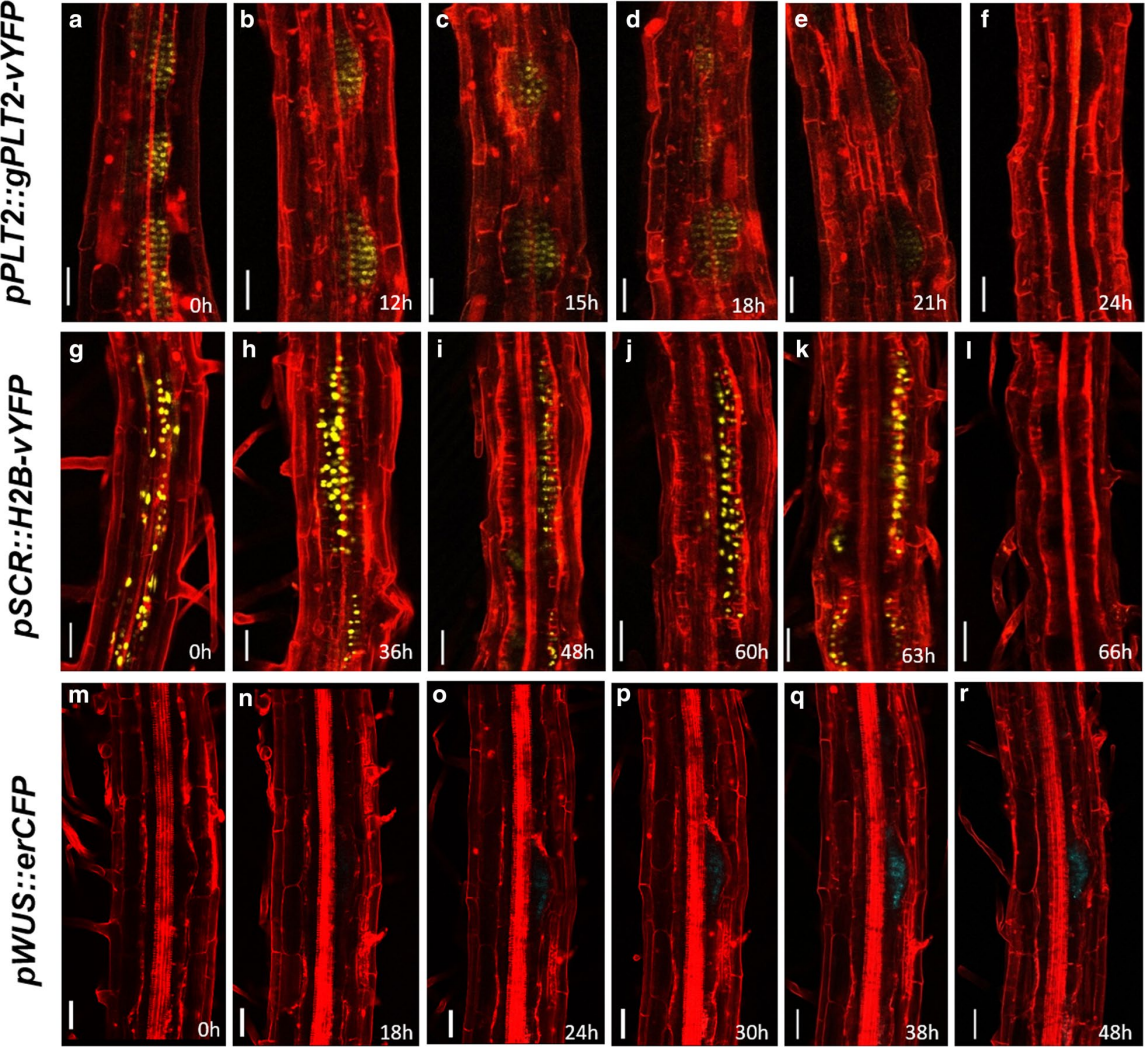
Confocal time lapse imaging of hourly resolution during LRP to shoot transition. **a**–**f** Time-lapse imaging showing *PLT2*-*vYFP* expression during the early hours of LRP to shoot conversion. **f** PLT2-vYFP expression is undetectable at 24 h on DSIM. **g**–**l** Gradual disappearance of *pSCR::H2B*-*vYFP* expression upon DSIM treatment. **l** 66 h of DSIM treatment leads to the loss of *pSCR::H2B*-*vYFP* expression from LRP. **m**–**r** Induction of *pWUS::erCFP* on 24 h of DSIM treatment. *Red signal* is propidium iodide stain. *Scale bar* 50 µm

Cytokinin triggers massive reprogramming associated with *trans*-differentiation. Studying the spatio-temporal expression patterns of cell fate specific regulators using confocal time-lapse imaging would enable to understand the early molecular events during *trans*-differentiation. Our method illustrates how time-lapse imaging could be revealing to study the process of conversion of LRP to shoot. We have used a set of cell fate specific fluorescent markers to analyze their expression patterns. We observed significant changes in the expression patterns of these cell fate specific markers even before morphological changes were visible in LRP. First we monitored the expression pattern of root specific stem cell regulator, PLETHORA2 (PLT2) [23] tagged with yellow fluorescent protein (YFP) during the conversion of LRP into shoot. The translational fusion *pPLT2::PLT2*-*vYFP* was expressed in stem cell niche of primary root meristem and in LRP from stage II onwards as previously reported [13, 15]. Time-lapse imaging showed the drastic reduction in *PLT2*-*vYFP* expression in LRP soon after transferring to DSIM and it was undetectable after 24 h, suggesting the rapid cell fate changes (Figs. 5b, 6a–f). As expected, PLT2-vYFP expression remained absent in the nascent shoot meristem and leaf primordia (Fig. 5c, d). Next we examined the expression pattern of QC and endodermis specific *SCARECROW* (*SCR*) gene [24, 25] during LRP-to-shoot conversion. Before transferring to DSIM, *pSCR::H2B*-*vYFP* was expressed in LRP (Figs. 5e, 6g). *pSCR::H2B*-*vYFP* expression was detectable even after 63 h of DSIM treatment (Figs. 5f, 6h–k) but its expression was down regulated afterwards (Figs. 5g, 6l). *pSCR::H2B*-*vYFP* expression was reinstated in regenerating leaves at petiole and mid-vein. Next, we analyzed the spatio-temporal expression pattern of shoot meristem specific marker, SHOOT MERISTEMLESS (STM) [26] during the LRP-to-shoot conversion. Expression of *pSTM::STM*-*vYFP* was neither detectable in untreated LRP nor in LRP at early days on DSIM (Fig. 5i–k). The earliest *pSTM*-*vYFP* expression was noticed when the shoot meristem developed from LRP (Fig. 5l). We next examined the expression pattern of shoot stem cell regulator, WUS [27] during the conversion. *pWUS::erCFP* was expressed in the organizing center of shoot apical meristem and not in LRP as previously reported (Fig. 5m) [6]. Upon DSIM treatment, *pWUS::erCFP* expression was rapidly induced in LRP (Figs. 5n, o, 6o–r). Once the shoot meristem is formed, expression of *pWUS::erCFP* got confined to a smaller domain of shoot meristem as reported in our previous study (Fig. 5p) [6]. We noted the *pWUS::erCFP* expression in treated LRP (at 24 h) much prior to the disappearance of *pSCR::H2B*-*vYFP* (at 66 h) suggesting the intermediate developmental phases during LRP-to-shoot conversion (Figs. 5f, n, 6l, o). It is important to note that the onset of p*STM:STM*-*vYFP* expression is much delayed as compared to *pWUS::erCFP* during this process (Figs. 5l, n, 6o). Together, the expression pattern of tissue specific cell fate markers for root and shoot clearly demonstrate dynamic changes in the relative abundance of stem cell regulators and rapid cell fate changes during the conversion.

**Fig. 7:**
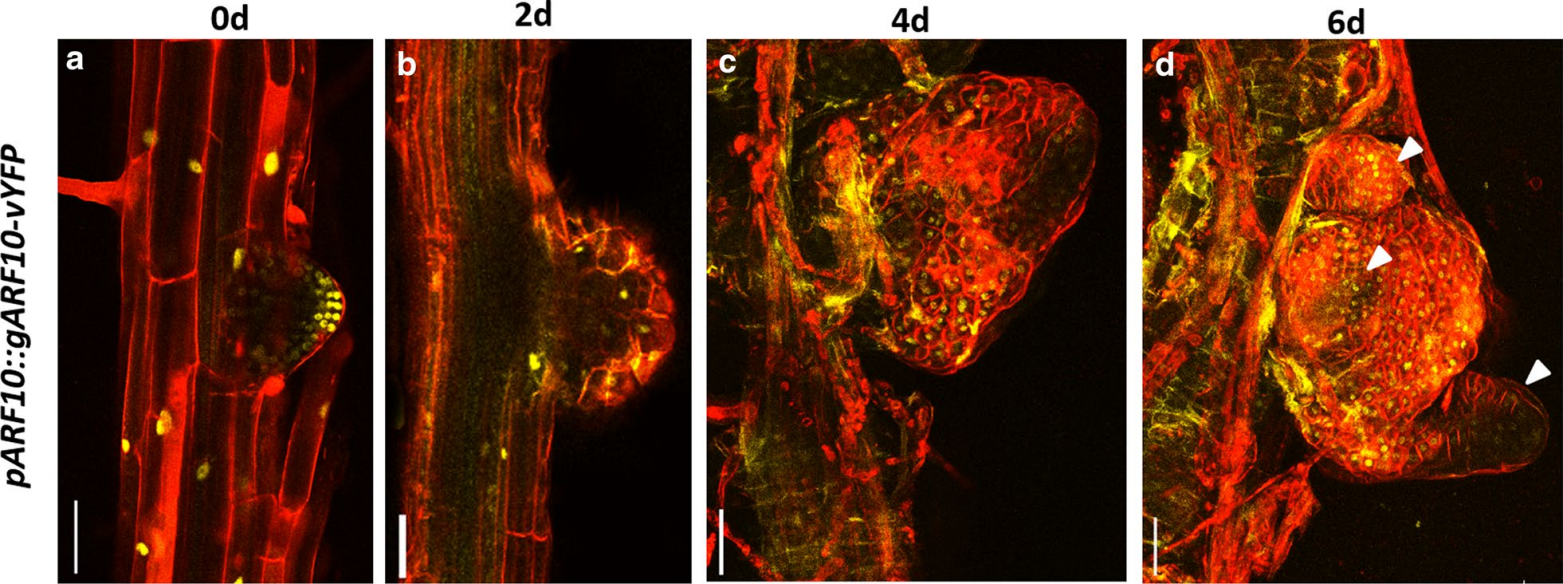
Time lapse imaging of auxin response factor during conversion. **a**–**d** Confocal time-lapse imaging showing *ARF10*-*YFP* expression during the conversion of LRP to shoot. Note the persistence of *ARF10*-*YFP* expression in both LRP and leaf primordia. Red signal is propidium iodide stain in (**a**, **b**) and FM4-64 stain in (**c**, **d**). *Scale bar* 50 µm

Further we analyzed the spatiotemporal expression pattern of the factors involved in auxin signaling pathway. *AUXIN RESPONSE FACTOR10* (*ARF10*) regulates the auxin mediated signal transduction pathway [28]. *pARF10::ARF10*-*vYFP* was expressed in untreated primary root meristem and LRP (Fig. 7a). In LRP its expression was predominantly detected in epidermis. Upon cytokinin treatment, *ARF10*-*vYFP* expression persisted in LRP undergoing fate changes, although its level was reduced (Fig. 7b, c). High level of *ARF10*-*vYFP* expression was observed in shoot meristem and leaf primordia (Fig. 7d).

In different plant species, the ideal culture conditions such as culture temperature, developmental stages of LRP and hormonal concentration required for the transition can vary. Based on our results in *Arabidopsis*, we recommend to standardize (1) temperature, (2) developmental stages of lateral root primordia (preferably early stages of LRP), (3) concentration of cytokinin, to adapt our protocol for other plant species.

Collectively, our method describes the use of time-lapse imaging technique for the fine dissection of the early developmental changes during *trans*-differentiation in *Arabidopsis*. We have also explored the role of external stimuli such as light and temperature in the LRP to shoot conversion. Further, this method may be adopted for other plant species on standardizing the parameters that influence the conversion process.

## Conclusion

Studies on the conversion of LRP-to-shoot are important in the light of the fact that many plant species adopt root-to-shoot conversion as one of the natural means of vegetative propagation. Many species like murraya, poplars, guava etc. have the natural capacity to make shoots from root when attached to or detached from the mother plant. The newly formed plant is called ‘a sucker’ or ‘a root sprout’ and these eventually grow as an independent plant. Although the frequency of sucker formation is less, some plant species mainly depend upon this mode of reproduction when seed based propagation is rare. Studies on this mode of propagation are highly fragmentary and the mechanisms underlying root-to-shoot conversion remain unknown. Although our method has been developed for *Arabidopsis*, it can be used as a reference to study the cellular and molecular mechanism underlying *trans*-differentiation in other plant species. Taken together, our method for the LRP-to-shoot conversion is highly efficient, independent of genotype tested and is amenable for time-lapse imaging to delineate the mechanism underlying this process.

## Additional files

10.1186/s13007-016-0127-5 Stage of LRP determines root to shoot conversion. (**A**) Late stage LRP of wild type plant on DSIM does not convert to shoot (**B**) Wild type untreated plant on ½ MS with outgrown lateral roots. Arrowhead in A marks outgrown lateral roots. Scale bar: 1 mm.
10.1186/s13007-016-0127-5 Effect of temperature on LRP to shoot conversion. Incubation at lower temperature (18 °C/20 °C cycle) marginally reduced the efficiency of LRP-to-shoot conversion as compared to 22 °C.
10.1186/s13007-016-0127-5 Effect of light on LRP to shoot conversion. Diurnal and continuous light conditions produced no significant difference in LRP to shoot conversion in explants (*p* > 0.05).
