# Supplementary material for: Protocol: a method to study the direct reprogramming of lateral root primordia to fertile shoots
Source: Plant Methods. 2016 May 12;12:27. doi: 10.1186/s13007-016-0127-5 (PMC4865056; doi:10.1186/s13007-016-0127-5)
Supplement: Supplementary file 1 — 10.1186/s13007-016-0127-5 Stage of LRP determines root to shoot conversion. (A) Late stage LRP of wild type plant on DSIM does not convert to shoot (B) Wild type untreated plant on ½ MS with outgrown lateral roots. Arrowhead in A marks outgrown lateral roots. Scale bar: 1 mm. [file 13007_2016_127_MOESM1_ESM.docx]

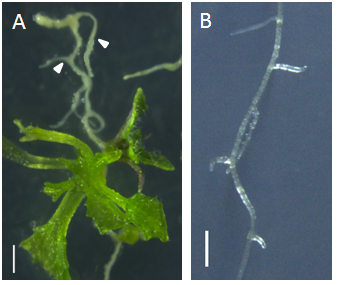


**Additional file 1: Stage of LRP determines root to shoot conversion (A)** Late stage LRP of wild type plant on DSIM does not convert to shoot **(B)** Wild type untreated plant on ½ MS with outgrown lateral roots. Arrowhead in A marks outgrown lateral roots. Scale bar: 1mm
