# Supplementary material for: Protocol: a method to study the direct reprogramming of lateral root primordia to fertile shoots
Source: Plant Methods. 2016 May 12;12:27. doi: 10.1186/s13007-016-0127-5 (PMC4865056; doi:10.1186/s13007-016-0127-5)
Supplement: Supplementary file 2 — 10.1186/s13007-016-0127-5 Effect of temperature on LRP to shoot conversion. Incubation at lower temperature (18 °C/20 °C cycle) marginally reduced the efficiency of LRP-to-shoot conversion as compared to 22 °C. [file 13007_2016_127_MOESM2_ESM.docx]

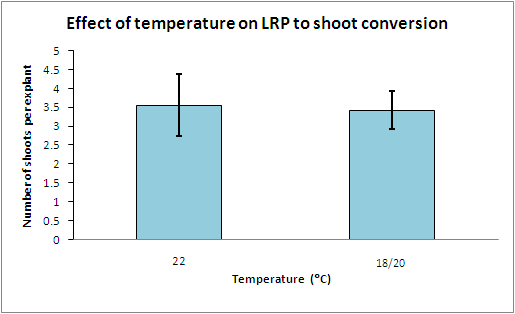


**Additional File 2: Effect of temperature on LRP to shoot conversion** Incubation at lower temperature (18 °C/20 °C cycle) marginally reduced the efficiency of LRP-to-shoot conversion as compared to 22 °C. Error bar represents standard error.
