# Supplementary material for: Protocol: a method to study the direct reprogramming of lateral root primordia to fertile shoots
Source: Plant Methods. 2016 May 12;12:27. doi: 10.1186/s13007-016-0127-5 (PMC4865056; doi:10.1186/s13007-016-0127-5)
Supplement: Supplementary file 3 — 10.1186/s13007-016-0127-5 Effect of light on LRP to shoot conversion. Diurnal and continuous light conditions produced no significant difference in LRP to shoot conversion in explants (p > 0.05). [file 13007_2016_127_MOESM3_ESM.docx]

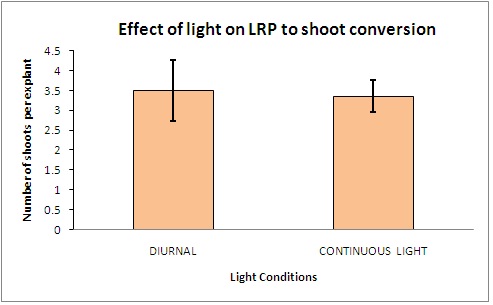


**Additional File 3: Effect of light on LRP to shoot conversion** Diurnal and continuous light conditions produced no significant difference in LRP to shoot conversion in explants. Error bar represents standard error (*p*>0.05)
